# Late-term abortion associated with *Trueperella abortisuis* in Austria: a case report combined with six years of retrospective diagnostic data

**DOI:** 10.1186/s12917-026-05818-9

**Published:** 2026-08-10

**Authors:** Lukas Schwarz, Christine Unterweger, Igor Loncaric, Rene Brunthaler, Heinrich Kreutzmann, Anja Joachim, Barbara Hinney, Andrea Buzanich-Ladinig

**Affiliations:** 1 University of Veterinary Medicine Vienna, Clinical Department for Farm Animals and Food System Transformation, Clinical Centre for Population Medicine in Fish, Pig and Poultry, Unit Swine Medicine, Vienna, Austria; 2 University of Veterinary Medicine Vienna, Department of Biological Sciences and Pathobiology, Centre of Pathobiology, Unit Microbiology, Vienna, Austria; 3 University of Veterinary Medicine Vienna, Department of Biological Sciences and Pathobiology, Centre of Pathobiology, Unit Pathology, Vienna, Austria; 4 University of Veterinary Medicine Vienna, Department of Biological Sciences and Pathobiology, Centre of Pathobiology, Unit Parasitology, Vienna, Austria; 5 MSD Animal Health, Intervet GesmbH, Vienna, Austria

**Keywords:** *Trueperella abortisuis*, Late-term abortion, Sow, Foetal bronchopneumonia, Placentitis

## Abstract

**Background:**

*Trueperella (T.) abortisuis* is an emerging Gram-positive bacterium increasingly associated with reproductive failure in pigs, yet its pathogenic role remains incompletely understood. This study describes a late-term abortion in a sow linked to *T. abortisuis* and integrates six years of retrospective diagnostic data from Austria.

**Case presentation:**

A clinically anorectic sow aborted eleven foetuses at gestational day 100 despite systemic antimicrobial treatment. *T. abortisuis* was isolated in high quantities from foetal stomach contents, umbilical cord blood, and amniotic fluid in most foetuses. Histopathology revealed severe suppurative bronchopneumonia in seven foetuses and necro-suppurative placentitis with intralesional Gram-positive bacteria in four placentae. Common viral and bacterial abortifacient agents were excluded by PCR. Retrospective analysis of routine diagnostics between 2019 and 2025 identified *T. abortisuis* in 31 samples from 14 farms, predominantly associated with genital tract specimens and abortion material, with consistently high bacterial loads in pooled foetal stomach contents.

**Conclusions:**

This report provides further evidence supporting the abortifacient potential of *T. abortisuis* in swine. The combination of characteristic suppurative and necrotizing placentitis, foetal infection, exclusion of common abortifacients, and supportive institutional diagnostic observations suggests that *T. abortisuis* should be considered in the differential diagnosis of porcine reproductive failure. The apparent lack of clinical response to systemic β-lactam treatment may reflect the advanced stage of foetal and placental infection at the time therapy was initiated, in addition to the limited knowledge regarding placental transfer of systemically administered antimicrobials in pregnant sows.

**Supplementary Information:**

The online version contains supplementary material available at https://doi.org/10.1186/s12917-026-05818-9.

## Background

*Trueperella (T.) abortisuis* is a Gram-positive, facultatively anaerobic bacterium which has received increasing attention in swine medicine over recent years. It was first described in Japan in 2009 as *Arcanobacterium abortisuis* after being isolated from a case of suppurative placentitis on a piglet-producing farm reporting intermittent abortions in sows [1]. In 2011, the organism was reclassified as a member of the genus *Trueperella* [2]. Since then, sporadic cases were reported from Germany, the United Kingdom, and the United States highlighting its emerging potential in the field of swine production and veterinary medicine [2–4]. While early reports primarily involved pigs or pig-associated specimens, *T. abortisuis* has more recently been identified in dogs and cats in non-reproductive clinical cases, as well as in a goat abortion case in which the significance of the bacterium as the primary cause remained uncertain [5, 6].

Since *T. abortisuis* has been described not only in cases of reproductive failure but also in clinically healthy animals, its exact role as a pathogen remains unclear. It is therefore currently considered a commensal or opportunistic bacterium [4]. Furthermore, there is limited information on the pathogenesis of *T. abortisuis* infections. However, the increasing number of case reports describing *T. abortisuis*-associated abortions suggests a potential causative effect, at least in pigs [7].

Lesions reported in previously published cases involving placentae and aborted foetuses were broadly similar. Macroscopically, described findings included meconium-stained foetuses with slight to moderate post mortem decomposition or obvious congenital abnormalities, increased amounts of fluid within body cavities, subcutaneous serosanguinous fluid, and haemorrhages affecting the umbilical cord and allantoic membrane [3, 8, 9]. Histopathological examination revealed suppurative bronchopneumonia, purulent to fibrinous omphalitis, and the presence of bacteria associated with these lesions [3, 8, 9].

The present case report adds novel evidence by combining detailed histopathological characterization of foetal and placental lesions with extensive bacteriological findings demonstrating high loads of *T. abortisuis* in multiple foetal compartments, including stomach contents, umbilical cord blood, and amniotic fluid. In addition, it integrates six years of retrospective routine diagnostic data from Austria, showing repeated detection of *T. abortisuis* in abortion material from multiple farms and thereby supporting its broader clinical relevance in porcine reproductive failure.

## Case presentation

In May 2022, a three-year-old Porcine Reproductive and Respiratory Syndrome Virus (PRRSV)-negative sow was purchased from the research and teaching farm of the University of Veterinary Medicine Vienna (Vetmeduni), Austria, for a research project at the Unit Parasitology. At the time of delivery, the sow was at gestational day 88, approximately 4 weeks antepartum. Upon arrival, the sow showed anorexia and was reluctant to stand up and move. Clinical examination four days after arrival revealed no deviations from the physiological norm, except for haemorrhagic contusions at the coronary band and the sole horn of the claws of both hind limbs that were painful on pressure. Transabdominal ultrasonography confirmed ongoing pregnancy with viable foetuses, indicated by foetal heartbeats. The vulva was slightly reddened without any vaginal discharge. Various types of feed were offered to motivate the sow to start feeding again, but all were refused. Rectal temperature remained within the normal range below 38.7 °C. However, a five-day treatment with meloxicam (0.4 mg/kg body weight) was initiated to alleviate suspected pain associated with locomotion.

On day six post-arrival (gestational day 94), the sow was still anorectic and developed vaginal discharge. Microbiological investigations of this discharge resulted in moderate quantities of *T. abortisuis* as the only culturable pathogen, which was confirmed by matrix-assisted laser desorption ionization-time-of-flight mass spectrometry (MALDI-TOF MS) (Bruker Daltonik, Bremen, Germany). Due to the lack of clinical breakpoints for *T. abortisuis*, antimicrobial susceptibility was interpreted using those established for *Streptococcus (S.) suis*, revealing full susceptibility to all tested β-lactam antibiotics, fluoroquinolones, phenicols, and sulphonamides. Consequently, antibiotic treatment with amoxicillin/clavulanic acid (8.75 mg/kg body weight) was initiated. Transabdominal ultrasonography still confirmed the viability of foetuses.

**Fig. 1:**
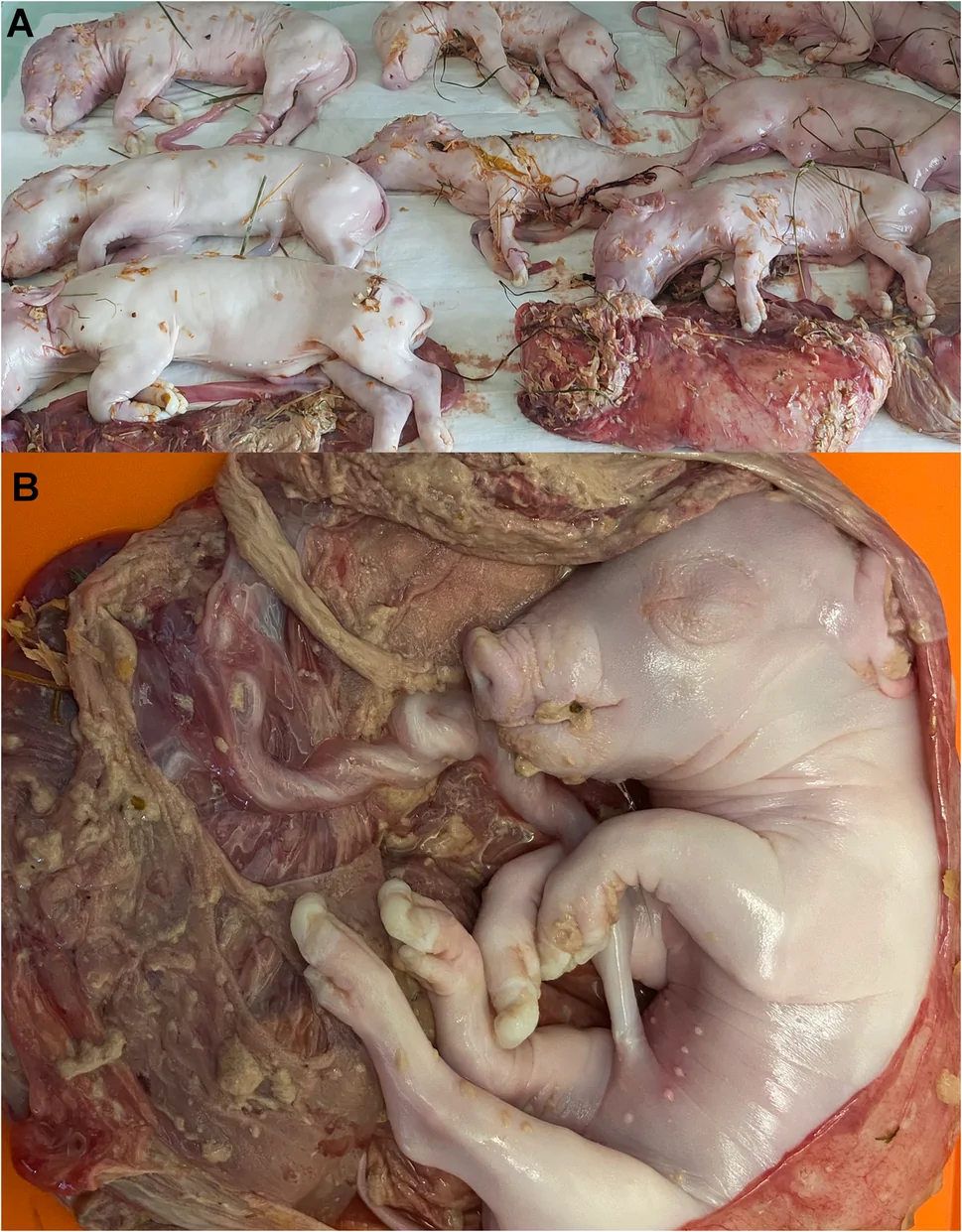
Fig. 1**A** shows the eight stillborn piglets delivered by the sow. Figure 1**B** represents an example of the grade of decomposition of a foetus. Furthermore, clear signs of necrotic placentitis are indicated by the light brownish material covering in parts also the foetus

The sow´s clinical condition did not show substantial improvement over the following days, and she continued to exhibit anorexia, while the body temperature was within the normal range. Twelve days after arrival at the experimental animal facilities of the Vetmeduni, the sow aborted on gestational day 100 and delivered eight stillborn piglets (Fig. 1A), some of which were partially or fully enclosed in foetal membranes. Following vaginal exploration and the subsequent intramuscular administration of 10 I.U. oxytocin, three additional stillborn piglets could be manually extracted. The complete aborted litter consisted of eleven piglets with two showing varying degrees of decomposition or slight autolysis (Fig. 1B). Clinically, the sow remained anorectic with a rectal temperature of 39.1 °C. Following the abortion, the required withdrawal period after treatment of the sow was observed, after which the sow was transported to an abattoir for slaughter. Within this period of observation, the clinical state of the sow remained clinically unsuspicious.

Given the previous microbiological identification of *T. abortisuis* in the vaginal discharge, a potential involvement of this organism in the late-term abortion was suspected. Consequently, targeted microbiological and pathological investigations were initiated on the aborted foetuses to assess the presence and possible etiological role of *T. abortisuis*. More specifically, foetuses were necropsied following a simplified protocol used at Unit Swine Medicine, University of Veterinary Medicine Vienna, Austria. Each foetus was necropsied using sterile scissors, scalpel and forceps. First the thorax was opened, and a specimen of the thymus was collected for PRRSV reverse transcriptase (RT) polymerase chain reaction (PCR) and a specimen of the heart for Porcine Circovirus (PCV) 2/PCV3 PCR. For the detection of *T. abortisuis* at least lung tissue, stomach content, and umbilical cord swabs were collected as well as placenta, if assignable to a foetus. Each sample was taken individually. For *Leptospira* spp. and *Chlamydia* spp., pooled samples consisting of lung, liver, kidney and stomach content were collected individually from each foetus. All foetuses were inspected macroscopically including assessment of the airways and main organs, representing a common condition of aborted foetuses: non-ventilated foetal lungs and absence of gross lesions, except for advanced decomposing state in one foetus. Pathogens causing stillborn foetuses and abortions known to occur frequently in Austria – PRRSV, PCV2, PCV3, leptospira and chlamydia - were investigated but not detected by PCR. The crown-rump-length of each foetus was measured (Table 1) and all foetuses were necropsied individually under a laminar flow using sterile equipment for each foetus to prevent cross contamination.

**Table 1:** Microbiologic results of the investigated foetal material. Grading refers to the detection of *T. abortisuis.* CRL= crown-rump length, +++= high bacterial load, ND = not detected, NI = not investigated

Foetus	CRL in mm	Stomach content	Umbilical cord	Amniotic fluid
1	275	+++	ND	NI
2	255	+++	ND	
3	250	+++	ND	
4	300	+++	ND	
5	250	+++	+++	
6	240	ND	ND	
7	200	+++	+++	
8	240	+++	+++	
9	270	+++	ND	+++
10	240	+++	ND	NI
11	270	ND	+++	

### Microbiological investigations

Individual specimens included stomach content aspirated by sterile syringe and needle, a swab of the blood of the umbilical cord and in case of piglet 9 also aspiration of the clearly assignable amniotic fluid. Selection of the specimens to be investigated was based on the successful isolation given in previous reports [3, 10]. Samples were processed following standard cultivation protocols for cultivable Gram-positive and Gram-negative bacteria. *Trueperella abortisuis* was isolated in high quantities from nine out of eleven stomach content samples and from four out of eleven umbilical cord swabs and species discrimination was determined using MALDI-TOF MS (Table 1). Furthermore, a high bacterial load of *T. abortisuis* was isolated in the amniotic fluid of piglet 9. *Trueperella abortisuis* could be isolated from all but one piglet. Further isolated bacteria included low amounts of members of the Enterobacterales and single colonies of staphylococci and *Mammaliicoccus sciuri.*

### Pathologic investigations

**Fig. 2:**
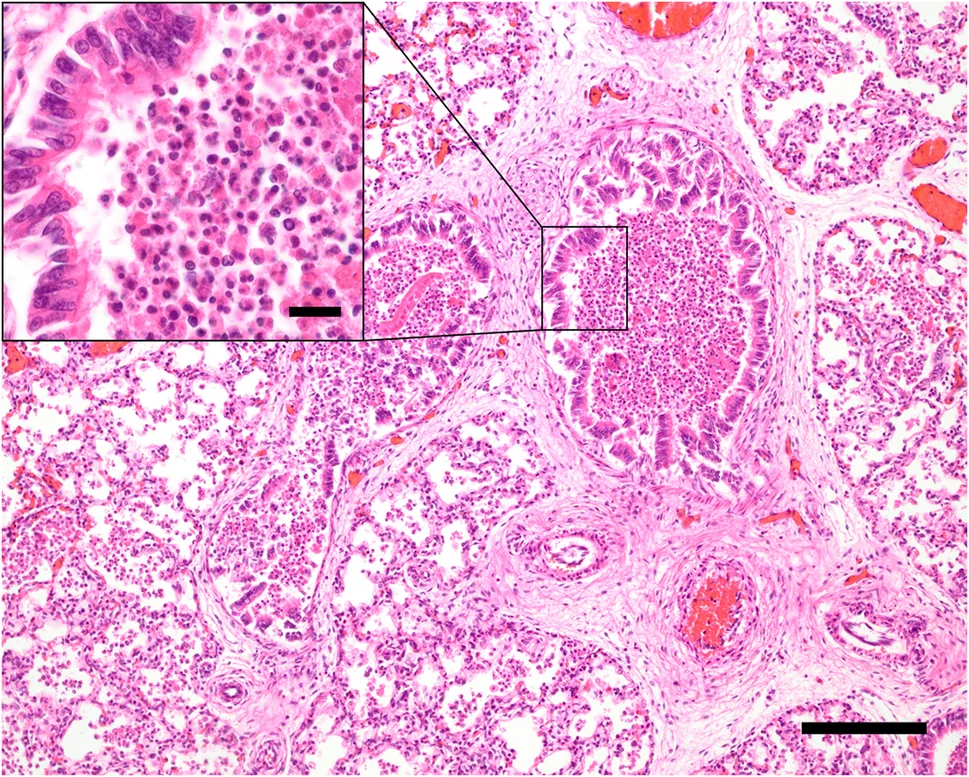
Lung of an aborted piglet (H&E stain) with changes consistent with severe suppurative bronchopneumonia and mild interstitial oedema and atelectasis. Bronchioles are filled with large amounts of neutrophils. The bronchiolar epithelium is markedly desquamated. Scale bar = 160 μm; Inset: Higher magnification of a bronchiole demonstrating abundant intraluminal suppurative exudate composed predominantly of neutrophils. Scale bar = 20 μm

**Fig. 3:**
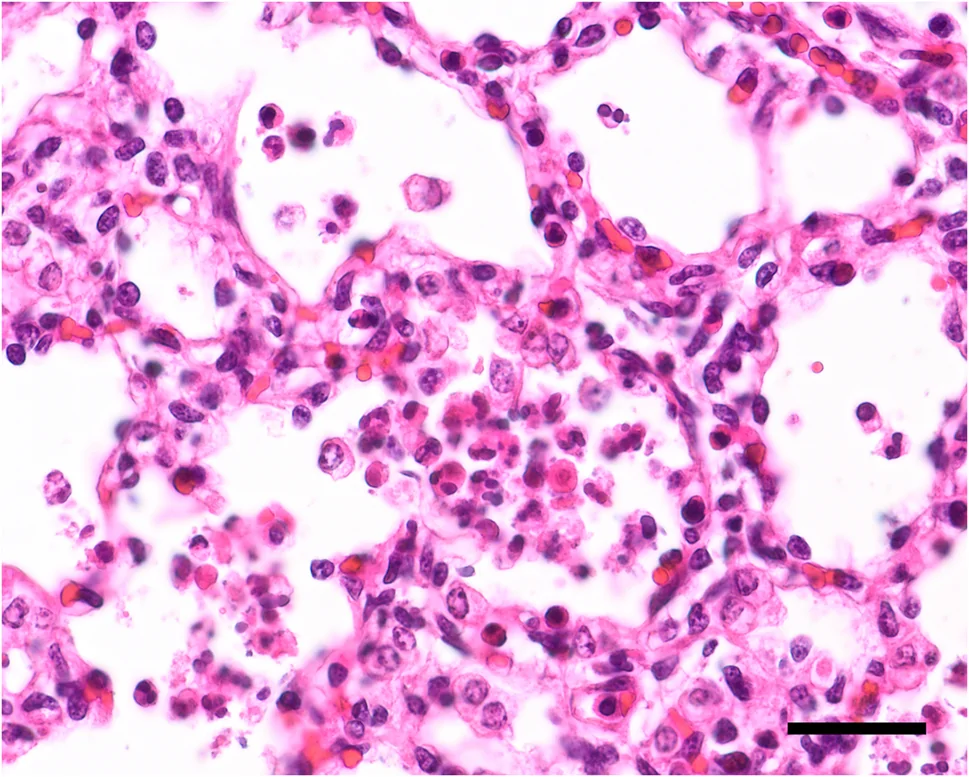
A higher-magnification view of the foetal lung from an abortion case, showing numerous intra-alveolar neutrophils and some macrophages, as well as marked hyperaemia (H&E stain). Scale bar = 30 µm

For histopathologic investigations heart, lung, kidney, liver, spleen, stomach, umbilical cord and if assignable also placental tissue were collected and fixed in 10% neutral buffered formalin. Following adequate fixation, tissues were routinely processed and embedded in paraffin. Paraffin sections were prepared and subjected to standard haematoxylin–eosin (H&E) staining. Stained slides were examined by light microscopy. The lungs of 7 out of 11 piglets showed significant histopathologic changes. The lung parenchyma showed mild to moderate interstitial oedema. Scattered neutrophils were present within the interstitium, and the pulmonary capillaries were markedly congested. Several bronchi and bronchioles were filled with abundant suppurative exudate consisting mainly of degenerated and non-degenerated neutrophils admixed with cellular debris, while the alveolar lumina contained numerous neutrophils and occasional macrophages (Figs. 2 and 3). In addition, individual bronchioles contained keratin debris and meconium, consistent with intrauterine aspiration of amniotic fluid; however, there were no signs of pulmonary ventilation. As expected, foetal atelectasis was observed; this is a physiological phenomenon in non-ventilated foetal lungs and was therefore not considered a pathological finding. Only a few extracellular short rod-shaped bacteria were observed, occurring singly or in small aggregates within the suppurative exudate. Overall, the findings were consistent with acute suppurative bronchopneumonia and aspiration of amniotic fluid and meconium (Table 2).

**Table 2:** Overview of the major and consistent histopathologic results of investigated foetal and placental tissues. SBP= suppurative bronchopneumonia, SNP= suppurative and necrotic placentitis, NPA = no pathologic abnormalities, NI = not investigated

Foetus	Histopathologic findings of foetal lung	Histopathologic findings of placenta
1	NPA	NI
2	Early SBP	NI
3	Fluid/meconium aspiration	NI
4	SBP	Severe multifocal, SNP
5	SBP	NI
6	Early SBP	NI
7	Early SBP	Severe multifocal to confluent, SNP
8	SBP	Severe focal, SNP
9	NPA	moderate, oligofocal SNP
10	SBP	NI
11	Fluid/meconium aspiration	NI

**Fig. 4:**
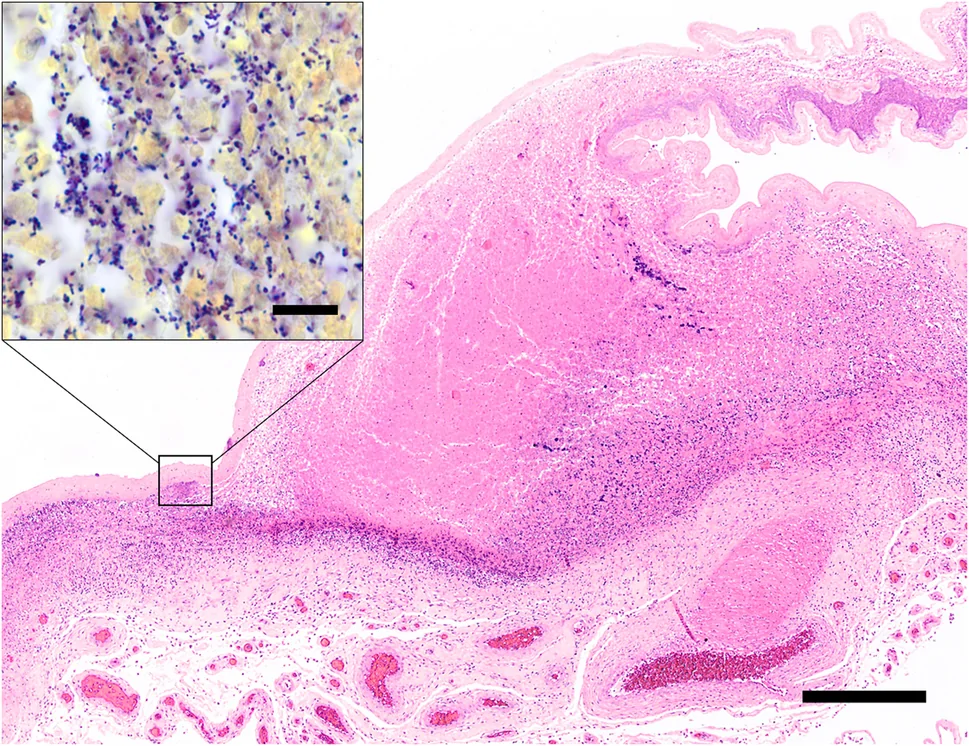
Severe necro-suppurative placentitis with large amounts of intralesional bacterial microcolonies and severe hyperaemia (H&E stain). Scale bar = 400 µm; Inset: Taylor's Gram stain demonstrating numerous intralesional, purple-black, short rod-shaped bacteria consistent with the morphology of Trueperella abortisuis. Scale bar = 10 µm

In addition, four placentae that could be assigned to individual piglets showed significant histopathologic changes. The examined placental tissue represented the chorioallantois and exhibited severe, diffuse oedema affecting the full thickness of the chorioallantoic stroma, resulting in marked expansion and separation of the stromal tissue. The remaining placentae were not investigated because they could not be reliably assigned to the corresponding foetuses. Pronounced diffuse hyperaemia accompanied the oedema. Additionally, multifocal, severe suppurative and necrotizing placentitis was present (Fig. 4; Table 2). The inflammatory lesions predominantly affected the subepithelial chorioallantoic stroma and were associated with multifocal accumulations of fibrinosuppurative exudate on the chorioallantoic surface. The inflammatory infiltrates consisted mainly of numerous neutrophils, frequently forming dense aggregates within the affected areas, and were associated with marked tissue necrosis. Numerous intralesional bacteria were present within the necrotic and inflammatory foci. These bacteria appeared as abundant short rod-shaped organisms, often arranged in clusters and embedded within necrotic debris and neutrophilic exudate. Overall, the findings were consistent with severe, acute, necrotizing bacterial chorioallantoitis. Bacteria identified in the haematoxylin–eosin (H&E)-stained sections were further characterized using Taylor’s method for Gram staining on paraffin sections to determine their Gram reaction [11]. Using this staining technique, numerous purple-black stained microorganisms were identified, suggesting the presence of Gram-positive bacteria (inset Fig. 4). Bacteria consistent with the rod-shaped morphology of *T. abortisuis* were identified within tissue lesions during histopathological examination.

The remaining examined tissues (heart, liver, spleen, kidneys, stomach, and umbilical cord) did not show any significant histopathological alterations.

### Descriptive routine diagnostic observations from 2019 to 2025 at Vetmeduni

In routine diagnostics at Vetmeduni, T. abortisuis was first identified in 2019 from a bacterial enrichment culture of a vaginal swab collected from a sow. In subsequent years, T. abortisuis was sporadically cultured from reproductive tract-associated samples and abortion material submitted for routine diagnostic examination. Because these observations originated from non-systematic diagnostic submissions with variable sample types and incomplete case information, they are presented descriptively in Supplementary File 1.

## Discussion and conclusion

This case highlights a late-term abortion in a sow strongly associated with *T. abortisuis* infection and provides further evidence for the pathogenic potential of this emerging bacterium. The lack of clinical response to systemic antimicrobial treatment may reflect that foetal infection was already advanced at the time therapy was initiated. Causality is strongly supported by the isolation of *T. abortisuis* in high abundance from multiple foetuses, umbilical cord blood, and amniotic fluid, together with characteristic necro-suppurative placentitis containing intralesional Gram-positive bacteria, consistent foetal suppurative bronchopneumonia, and exclusion of common infectious abortifacients. Taken together, these findings indicate *T. abortisuis* as the most likely etiological agent rather than post-abortion contamination.

The histopathological lesions observed in this case, including suppurative bronchopneumonia in 7 of 11 foetuses and multifocal necro-suppurative placentitis in four placentae, are in line with previous reports of *T. abortisuis*-associated abortions [8, 9]. Similar lesions have been described in cases from other countries, and our findings further extend the geographic evidence for the association of *T. abortisuis* with porcine reproductive failure [1, 3, 10]. Other bacteria were cultivated only sporadically and in low abundance, supporting their interpretation as incidental contaminants rather than primary pathogens. The pathogenic potential of *T. abortisuis* remains a subject of ongoing investigation. While initially described based on a single strain isolated from an aborted sow’s placenta in Japan [1], its classification as a primary pathogen remains under debate, largely due to occasional co-isolation with other bacteria from both diseased and clinically healthy animals. Some authors have suggested that *T. abortisuis* could be an opportunistic pathogen of the urogenital tract in pigs and other animals [4, 5, 12]. However, the consistent isolation of *T. abortisuis* as the dominant organism in the present porcine case and in other reported abortion cases, together with characteristic inflammatory lesions in examined swine cases, supports its potential role as an abortifacient pathogen. A caprine abortion case has also been reported, although the diagnostic evidence in that instance was more limited, since no foetal and/or placental material was investigated [6]. Additional evidence comes from its detection in boar semen in the United States without clinical signs in the boars, in the prepuce of a boar in Scotland, and in a case of seminal vesiculitis in a boar, demonstrating its broader presence in the male reproductive tract [4, 10, 13, 14]. Additionally, it has been isolated from other animal species, including cows with vaginitis and metritis, as well as in companion animals such as dogs and cats suffering from various abscesses or urinary tract infections [5, 15].

In our institutional experience, *T. abortisuis* has been sporadically detected in reproductive tract and abortion-associated samples; however, these observations derive from non-systematic diagnostic submissions and should be interpreted cautiously (see Supplementary File 1 for descriptive details). Alternative routes of infection should also be considered. Although ascending infection from the maternal genital tract remains a plausible mechanism, haematogenous dissemination following transient bacteraemia cannot be excluded, particularly as transport and relocation shortly before the onset of clinical signs may have contributed to stress-associated immune impairment of the sow. In addition, the natural reservoir of *T. abortisuis* remains incompletely understood, but current evidence suggests that colonization of the genital mucosa in both female and male pigs may serve as a potential source of infection. Of particular interest in the present case is the persistent high bacterial load of *T. abortisuis* in foetal tissues and fluids despite systemic antimicrobial treatment of the dam with amoxicillin-clavulanic acid. In vitro susceptibility based on *Streptococcus suis* clinical breakpoints indicated full sensitivity to β-lactams. However, in vivo efficacy was absent. This discrepancy raises important questions regarding the efficacy of antimicrobial therapy in treating genital infections in pregnant sows. One of the reasons for treatment failure lies in the physiological characteristics of the porcine placenta which is of epitheliochorial type and consists of six different layers, limiting the transfer of large molecules [16, 17]. The transplacental transfer of antimicrobial agents is highly dependent on molecular characteristics such as lipophilicity and molecular weight. Amoxicillin, being rather hydrophilic than lipophilic likely exhibits poor placental permeability [18]. Assuming that amoxicillin and clavulanic acid are too large molecules to be sufficiently transferred to the foetuses *in utero* when treating the sow systemically, we hypothesize that no or only very low concentrations of amoxicillin/clavulanic acid reached the foetuses *in utero* to exert a therapeutic effect. Although this hypothesis is biologically plausible, controlled pharmacokinetic studies in pregnant sows are currently lacking. The reported case therefore raises important questions regarding the effectiveness of systemic antimicrobial treatment for genital infections in pregnant sows and highlights the lack of data on antimicrobial pharmacokinetics and pharmacodynamics in porcine foetuses. Such data are urgently needed to guide evidence-based treatment strategies. Another possible explanation is that foetal infection with *T. abortisuis* was already well established when treatment was initiated, making prevention of abortion unlikely. If insufficient concentrations of amoxicillin reached the foetuses, the infection may have continued to progress, resulting in foetal death, loss of the foetal endocrine signals required to maintain pregnancy, and subsequent abortion.

This case highlights the diagnostic challenges posed by emerging pathogens such as *T. abortisuis* lacking the fulfilment of Koch´s postulates. Its lack of recognition in past abortion cases may be attributed to limited pathogenesis data and inconsistent detection. The lack of *T. abortisuis* growth in one foetus from this litter further highlights variability in foetal infection and host susceptibility. Comprehensive diagnostic workups, including bacterial culture, histopathology, and molecular techniques, are essential to confirm *T. abortisuis* involvement and to exclude other common infectious causes of abortion. Moreover, it is crucial to evaluate entire aborted litters or, at minimum, pooled samples, to avoid overlooking a potential involvement of *T. abortisuis*. During this case, collection of diagnostic specimens happened under unusual clean conditions as aborted foetuses were not really contaminated with faecal material, as commonly encountered under field conditions. This was most probably the reason, why *T. abortisuis* solely could be isolated from the abortion material without any secondary bacterial cross-contamination.

A limitation of this case report is that the diagnostic work-up was performed under field-like, opportunistic conditions rather than as a prospective experimental study. Consequently, microbiological investigation focused on selected specimens, mainly foetal stomach contents, umbilical cord swabs and, in one foetus, amniotic fluid, and not all potentially informative tissues and fluids were available from every foetus. Therefore, the distribution and route of spread of *T. abortisuis* within individual foetuses could not be fully reconstructed.

In summary, this case adds to the growing number of reports supporting the abortifacient potential of *T. abortisuis*. Based on the accumulated evidence, we strongly recommend including *T. abortisuis* in the list of differentials when investigating reproductive failure or abortion of sows. Continued surveillance and research are particularly important to better understand the pathogenic potential of *T. abortisuis*, its interactions with other reproductive tract bacteria, and its role in disease reproducibility in swine populations.

## Supplementary Information

Supplementary Material 1: Supplementary File 1. Descriptive summary of routine diagnostic submissions to Vetmeduni in which Trueperella abortisuis was cultured between 2019 and 2025.

## Data Availability

All data generated and analysed during this case report are included in this article and its supplementary material.

## Abbreviations

CRL: Crown–rump length
GD: Gestational day
H&E: Haematoxylin and eosin
MALDI: TOF MS–Matrix–assisted laser desorption/ionization time–of–flight mass spectrometry
PCV2: Porcine circovirus type 2
PCV3: Porcine circovirus type 3
PCR: Polymerase chain reaction
PK/PD: Pharmacokinetics/pharmacodynamics
PRRSV: Porcine reproductive and respiratory syndrome virus
S. suis: Streptococcus suis
T. abortisuis: Trueperella abortisuis
Vetmeduni: University of Veterinary Medicine Vienna

## References

[1] Azuma R, Murakami S, Ogawa A, Okada Y, Miyazaki S, Makino T. *Arcanobacterium abortisuis* sp. nov., isolated from a placenta of a sow following an abortion. Int J Syst Evol Microbiol. 2009;59:1469–73. 10.1099/ijs.0.004465-0. 19502337

[2] Yassin AF, Hupfer H, Siering C, Schumann P. Comparative chemotaxonomic and phylogenetic studies on the genus *Arcanobacterium* Collins et al. 1982 emend. Lehnen et al. 2006: proposal for *Trueperella* gen. nov. and emended description of the genus *Arcanobacterium*. Int J Syst Evol Microbiol. 2011;61:1265–74. . 10.1099/ijs.0.020032-0 20622055

[3] Jørgensen PK, Ahmed MFE, Thomson J, Lämmler C, Bell J, Alssahen M, et al. First porcine abortion associated with *Trueperella abortisuis* in the United Kingdom. Vet Rec Case Rep. 2021. 10.1002/vrc2.129.

[4] Bussalleu E, Althouse GC. Identification of *Trueperella abortisuis* contamination in extended boar semen. J Swine Health Prod. 2017;25:299–302.

[5] Wickhorst J-P, Hassan AA, Sammra O, Alssahen M, Lämmler C, Prenger-Berninghoff E, et al. First report on the isolation of *Trueperella abortisuis* from companion animals. Res Vet Sci. 2019;125:465–7. 10.1016/j.rvsc.2017.11.003. 29132685

[6] Ghosh K, McGregor G, Hughes G, Trimble M, Hsiao W, Trokhymchuk A. First isolation and whole-genome sequencing of *Trueperella abortisuis* from a goat in Canada. Can Vet J. 2024;65:698–702. PMC11195495 38952754

[7] Alssahen M, Hassan AA, Wickhorst J-P, Sammra O, Lämmler C, Glaeser SP, et al. Epidemiological analysis of *Trueperella abortisuis* isolated from cases of pig abortion of a single farm. Folia Microbiol (Praha). 2020;65:491–6. 10.1007/s12223-019-00753-9. 31664640

[8] Ford A, Palinski R, Lubbers B, Tokach L, Cino-Ozuna AG. Placentitis and abortion in domestic pigs (*Sus scrofa domesticus*) associated with *Trueperella abortisuis* on US swine farms. J Swine Health Prod. 2022;30:95–100. 10.54846/jshap/1258.

[9] Murakami S, Ogawa A, Azuma R, Ohba T, Murata R. Aborted lesions of a pig associated with *Arcanobacterium abortisuis* and the immunohistochemical features. J Vet Med Sci. 2011;73:797–9. 10.1292/jvms.10-0469. 21224529

[10] Metzner M, Erhard M, Sammra O, Nagib S, Hijazin M, Alber J, et al. *Trueperella abortisuis*, an emerging pathogen isolated from pigs in Germany. Berl Münch Tierärztl Wschr. 2013;423–6. 10.2376/0005-9366-126-423. 24199385

[11] Taylor RD. Modification of the Brown and Brenn gram stain for the differential staining of gram-positive and gram-negative bacteria in tissue sections. Am J Clin Pathol. 1966;46:472–4. 10.1093/ajcp/46.4.472. 4162586

[12] Ulbegi-Mohyla H, Hassan AA, Hijazin M, Alber J, Lämmler C, Abdulmawjood A, et al. Characterization of *Arcanobacterium abortisuis* by phenotypic properties and by sequencing the 16S-23S rDNA intergenic spacer region. Vet Microbiol. 2011;148:431–3. 10.1016/j.vetmic.2010.08.026. 20926209

[13] Scotland’s Rural College Veterinary Services. *Trueperella abortisuis* causing abortion in pigs in Scotland. Vet Rec. 2019;185:162–5. 10.1136/vr.l5083. 31395715

[14] Savitske B, White B, Rogovskyy A, Washburn K. Seminal vesiculitis in a Vietnamese pot-bellied boar. Vet Rec Case Rep. 2021. 10.1002/vrc2.213.

[15] Hijazin M, Ulbegi-Mohyla H, Alber J, Lämmler C, Hassan AA, et al. Identification of *Arcanobacterium* (*Trueperella*) *abortisuis*, a novel species of veterinary importance, by Matrix-Assisted Laser Desorption Ionization-Time Of Flight mass spectrometry (MALDI-TOF MS). Berl Münch Tierärztl Wschr. 2012;32–7. 10.2376/0005-9366-125-32. 22372322

[16] Karniychuk UU, Nauwynck HJ. Pathogenesis and prevention of placental and transplacental porcine reproductive and respiratory syndrome virus infection. Vet Res. 2013;44:95. 10.1186/1297-9716-44-95. PMC4021427 24099529

[17] Leiser R, Kaufmann P. Placental structure: in a comparative aspect. Exp Clin Endocrinol. 1994;102:122–34. 10.1055/s-0029-1211275. 7995333

[18] Zaręba-Szczudlik J, Romejko-Wolniewicz E, Lewandowski Z, Różańska H, Czajkowski K. Concentration of amoxicillin in maternal serum, cord blood, amniotic fluid and the placenta after vaginal administration. J Matern Fetal Neonatal Med. 2015;28:2048–52. 10.3109/14767058.2014.976548. 25316563

