# Supplementary material for: Late-term abortion associated with Trueperella abortisuis in Austria: a case report combined with six years of retrospective diagnostic data
Source: BMC Vet Res. 2026 Aug 10;22:689. doi: 10.1186/s12917-026-05818-9 (PMC13757104; doi:10.1186/s12917-026-05818-9)
Supplement: Supplementary file 1 [file 12917_2026_5818_MOESM1_ESM.docx]

Supplementary file 1: Overview of positive detections of *T. abortisuis* in routine diagnostics of porcine specimens from 2019-2025. Each sow or weaner ID represents samples of individual animals. Low-grade detection was mainly observed in genital tract swabs, whereas abortion material consistently showed moderate to high bacterial loads of *T. abortisuis*, supporting a transition from colonization to clinically relevant intrauterine infection. GD: gestation day.

| **ID** | **Year** | **Annual abortion cases** | **Farm** | **Sample type** | **Production stage** | **Grade** | **Comments** |
| --- | --- | --- | --- | --- | --- | --- | --- |
| 1 | 2019 | 52 | 1 | Vaginal swab | Sow | after enrichment |  |
| 2 |  |  | 2 | Cervical swab | Sow | single colonies |  |
| 3 |  |  |  | Cervical swab | Sow | single colonies |  |
| 4 |  |  |  | Uterus mucosa | Sow | low to moderate | Genital tract necropsy |
| 5 |  |  |  | Uterus mucosa | Sow | low | Genital tract necropsy |
| 6 |  |  | 3 | Cervical swab | Sow | single colonies |  |
| 7 |  |  |  | Cervical swab | Sow | single colonies |  |
| 8 |  |  |  | Vaginal swab | Sow | single colonies |  |
| 9 |  |  |  | Vaginal swab | Sow | single colonies |  |
| 10 |  |  |  | Cervical swab | Sow | single colonies |  |
| 11 |  |  |  | Cervical swab | Sow | single colonies |  |
| 12 |  |  | 4 | Vaginal swab | Sow | moderate |  |
|  | 2020 | 50 | *T. abortisuis* not detected | | | | |
|  | 2021 | 53 |  |  |  |  |  |
| 17 | 2022 | 51 | 5 | Pooled stomach content | Foetuses | high | Abortion at GD 106 |
| 18 | 2023 | 47 | 6 | Pooled stomach content |  | high | Abortion at GD 100 |
| 19 |  |  | 7 | Pooled stomach content |  | high | Abortion at GD 81 |
| 20 |  |  | 8 | Serosal swab | Weaner | single colonies |  |
| 21 | 2024 | 64 | 7 | Pooled stomach content | Foetuses | high | Abortion at GD 112 |
| 22 |  |  | 9 | Cervical swab | Sow | single colonies |  |
| 23 |  |  | 10 | Uterus mucosa | Sow | high | Genital tract necropsy |
| 24 |  |  | 11 | Vaginal swab | Sow | moderate |  |
| 25 |  |  | 12 | Pooled stomach content | Foetuses | moderate | Abortion at GD 105 |
| 26 |  |  |  | Pooled stomach content | Foetuses | moderate |  |
| 27 |  |  | 13 | Vaginal swab | Sow | moderate |  |
| 28 |  |  |  | Cervical swab | Sow | high |  |
| 29 |  |  |  | Vaginal swab | Sow | moderate |  |
| 30 |  |  |  | Cervical swab | Sow | high |  |
| 31 | 2025 | 35 | 14 | Pooled stomach content | Foetuses | high | Abortion at GD 85 |

Routine diagnostics at Vetmeduni revealed *T. abortisuis* in 31 culture-positive samples originating from 14 different farms over a six-year period, indicating that the organism is geographically not only a rare pathogen confined to a single herd, but rather well distributed. In total in this time 352 cases associated with abortion in sows were investigated resulting in a proportion of *T. abortisuis*-related detection rate of 3.98 %. Importantly, 30/31 detections were associated with the female reproductive tract (vagina, cervix, uterus) or abortion material, while only a single isolate originated from a non-reproductive sample (serosal swab of a weaner). Interestingly, there was a clear difference in the detected grade during bacteriological cultivation. While vaginal and cervical swabs predominantly yielded single colonies or low–moderate growth, pooled foetal stomach contents from abortions consistently showed moderate to high bacterial loads, across multiple years (2022–2025) and farms, and were always linked to clinically evident reproductive failure (GD 81–112). Across the study period, *T. abortisuis* was cultivated from five abortion events from four farms, always in moderate to high abundance in pooled foetal stomach contents. This contrasts with the largely low-grade findings in routine vaginal/cervical swabs. Such a dichotomy supports the concept of *T. abortisuis* as a facultative pathogen - potentially present at low levels without clinical consequences, but capable of massive intrauterine proliferation once placental barriers are breached. The consistent recovery of high bacterial loads from aborted foetuses mirrors the present case report and aligns with published observations from other countries, reinforcing the hypothesis that placentitis as the consequence of ascending or haematogenous spread to the pregnant uterus represents a central pathogenic mechanism rather than post-abort contamination [1, 3, 4, 8, 13].

These data originate from non-systematic routine diagnostic submissions and should not be interpreted as prevalence or incidence data.
